# Multimodal Deep Learning Models for Detecting Dementia From Speech and Transcripts

**DOI:** 10.3389/fnagi.2022.830943

**Published:** 2022-03-17

**Authors:** Loukas Ilias, Dimitris Askounis

**Affiliations:** Decision Support Systems Laboratory, School of Electrical and Computer Engineering, National Technical University of Athens, Athens, Greece

**Keywords:** dementia, BERT, Log-Mel spectrogram, Vision Transformer, co-attention, Multimodal Shifting Gate, self-attention

## Abstract

Alzheimer's dementia (AD) entails negative psychological, social, and economic consequences not only for the patients but also for their families, relatives, and society in general. Despite the significance of this phenomenon and the importance for an early diagnosis, there are still limitations. Specifically, the main limitation is pertinent to the way the modalities of speech and transcripts are combined in a single neural network. Existing research works add/concatenate the image and text representations, employ majority voting approaches or average the predictions after training many textual and speech models separately. To address these limitations, in this article we present some new methods to detect AD patients and predict the Mini-Mental State Examination (MMSE) scores in an end-to-end trainable manner consisting of a combination of BERT, Vision Transformer, Co-Attention, Multimodal Shifting Gate, and a variant of the self-attention mechanism. Specifically, we convert audio to Log-Mel spectrograms, their delta, and delta-delta (acceleration values). First, we pass each transcript and image through a BERT model and Vision Transformer, respectively, adding a co-attention layer at the top, which generates image and word attention simultaneously. Secondly, we propose an architecture, which integrates multimodal information to a BERT model *via* a Multimodal Shifting Gate. Finally, we introduce an approach to capture both the inter- and intra-modal interactions by concatenating the textual and visual representations and utilizing a self-attention mechanism, which includes a gate model. Experiments conducted on the ADReSS Challenge dataset indicate that our introduced models demonstrate valuable advantages over existing research initiatives achieving competitive results in both the AD classification and MMSE regression task. Specifically, our best performing model attains an accuracy of 90.00% and a Root Mean Squared Error (RMSE) of 3.61 in the AD classification task and MMSE regression task, respectively, achieving a new state-of-the-art performance in the MMSE regression task.

## 1. Introduction

Alzheimer's disease (AD) is the most common form of dementia and may contribute to 60–70% of cases. According to the World Health Organization, approximately 55 million people suffer from dementia nowadays, while this number is going to present a surge in the upcoming years reaching up to 78 million and 139 million people in 2030 and 2050, respectively (World Health Organization, 2021). Due to the fact that Alzheimer's disease is a neurodegenerative disease, meaning that the symptoms become worse over time, the early diagnosis seems to be imperative for promoting early and optimal management. In addition, dementia is inextricably linked with difficulties in speech, since dementia affects how a person can use language and communicate (Alzheimer's Society, 2021). For this reason, current research works have moved their interest toward dementia identification from spontaneous speech, in order to save money and time.

Several research works have been proposed aiming to detect AD patients and predict their Mini-Mental State Examination (MMSE) scores using the modalities of both speech and transcripts. However, the majority of them have introduced label fusion and majority-voting or average approaches (Sarawgi et al., 2020; Chen et al., 2021; Syed et al., 2021). Specifically, regarding the AD classification task they train several textual and acoustic models and they make the final prediction of the given transcript based on the class, which received the most votes by the individual models. With regards to the MMSE regression task, they simply average the predictions of the individual models. Concurrently, they extract a large number of features corresponding to the textual and acoustic modalities and some of them train traditional machine learning algorithms, such as Logistic Regression, XGBoost, etc. The main limitation of feature extraction is the fact that it demands some level of domain expertise rendering it a time-consuming procedure. Thus, it is evident that these approaches are not time-efficient, since a lot of models must be trained and tested separately. At the same time, these approaches do not exploit the interaction between the two modalities. Moreover, research initiatives introducing multimodal models use the add and concatenation operation treating in this way equally the two modalities (Zhu et al., 2021). Another limitation of this approach has to do with the fact that one modality may override the other one with a negative impact on the classification performance. In addition, although transformers have achieved new state-of-the-art results in many domains, their potential has not been fully exploited yet to a high degree. Specifically, research works have exploited mainly pretrained transformer networks corresponding to the textual modality, such as BERT, RoBERTa, XLNet, etc.

In order to tackle the aforementioned limitations, we employ transformer-based networks, which can capture effectively the interaction between the different modalities and control the importance of each modality toward the final prediction. Compared with recent deep ensemble learning methods, which need to train models individually and then fuse the results of the classifiers, the proposed neural networks in this article can be trained in an end-to-end trainable manner. First, we extract Log-Mel spectrograms, their delta, and delta-delta (acceleration values) and construct an image per audio file consisting of three channels. Next, we introduce a neural network consisting of BERT and Vision Transformer (ViT) for extracting textual and visual embeddings, respectively, and add a co-attention mechanism over the respective embeddings, which can attend at the different modalities at the same time. In addition, we introduce an architecture, which integrates multimodal information into a BERT model *via* an Attention Gate called Multimodal Shifting Gate. To be more precise, we propose three variations of this architecture, where we inject *(a)* textual and visual, *(b)* textual and acoustic, and *(c)* textual, visual, and acoustic information into the BERT model. Finally, we propose an architecture, which can learn both the inter- and intra-modal interactions, i.e., image-image, text-text, text-image, and image-text, and show that it achieves state-of-the art results. Therefore, compared with prior works, our methods provide important advantages, since they can learn more representative features regarding the different modalities and require also less time for training.

Our main contributions can be summarized as follows:

- We conduct extensive experiments for detecting AD patients (AD classification task) and predicting the MMSE scores (MMSE regression task).
- We propose a multimodal model consisting of BERT, ViT, and a Co-Attention mechanism.
- We introduce an architecture, which incorporates a Multimodal Shifting Gate aiming to control the importance of text, acoustic, and visual representations. The conjunction of the textual, acoustic, and visual embeddings is fed to a BERT model.
- We propose an architecture aiming to model the inter- and intra-modal interactions of multimodal data.
- We achieve competitive results with state-of-the-art approaches on the ADReSS Challenge dataset both in the AD classification and MMSE regression task.
- Our best performing model achieves a new state-of-the-art result in the MMSE regression task.

## 2. Related Work

### 2.1. Unimodal Approaches

Chlasta and Wolk (2021) proposed a unimodal approach to detect AD patients only from speech. First, the authors used VGGish (Hershey et al., 2017) as a feature extractor followed by Principal Component Analysis (PCA). Finally, the authors trained support vector machines, linear support vector machines, perceptron, multi-layer perceptron classifier, and a k-NN classifier. Secondly, the authors introduced a convolutional neural network to detect AD patients using raw audio data and claimed that this approach outperformed the performance obtained *via* the traditional machine learning classifiers trained on VGGish features.

Meghanani et al. (2021a) introduced three deep neural networks, in order to explore the effectiveness of log-Mel spectrograms and MFCC features toward the detection of AD patients and the prediction of their MMSE scores. Specifically, they employed a convolutional neural network (CNN) followed by a long-short term memory network (CNN-LSTM), a pre-trained ResNet18 followed by LSTM, and a pyramidal Bi-LSTM followed by a CNN. Findings suggested that the incorporation of the log-Mel spectrograms and MFCCs features in deep neural networks are effective for detecting AD patients and predicting the MMSE scores.

Bertini et al. (2022) proposed an approach to detect AD patients using only speech data. More specifically, the authors extracted the log-Mel spectrogram for the audio files and trained an autoencoder, namely auDeep (Freitag et al., 2017). The authors passed the latent vector into a multilayer perceptron for classifying people into AD patients or non-AD ones.

Meghanani et al. (2021b) introduced some approaches to detect AD patients and predict the MMSE scores using only text data. Specifically, the authors proposed a Convolutional Neural Network (CNN) and fastText-based classifiers. Regarding the AD classification task, they fitted 21 models and the outputs were combined by a majority voting scheme for final classification. In terms of the MMSE regression task, the outputs of these bootstrap models were averaged for calculating the final MMSE score.

### 2.2. Multimodal Approaches

Chen et al. (2021) extracted a set of acoustic features, including GeMAPS, eGeMAPS (Eyben et al., 2015), etc., and a set of linguistic features, namely LIWC and BERT features. They fused the two modalities using early, late, and ensemble fusion strategies. A Logistic Regression classifier was exploited for classifying subjects in AD patients or not. Results suggested that average fusion of predicted class probabilities of the 10 best performing models achieved the highest accuracy accounting for 81.69%.

A similar approach was proposed by Syed et al. (2020), where the authors extracted a set of acoustic features, i.e., Prosody, Voice Quality, ComParE, IS10-Paling, etc., and a set of linguistic features using transformer-based networks, including BERT, RoBERTa, and their distilled versions. They categorized people into AD patients or not by training a Support Vector Machine (SVM) and a Logistic Regression (LR) classifier. The authors used label fusion from the top performing models and stated that the label fusion of the 10 best performing textual models achieved an accuracy of 85.42%. For predicting the MMSE scores, the authors used support vector machines based regression (SVR) and a partial least squares regressor (PLSR). They achieved a Root Mean Squared Error (RMSE) score equal to 4.30 by averaging the predictions of the MMSE scores from the top-10 performing models.

Similarly, Pompili et al. (2020) exploited an early fusion approach, in order to obtain a single feature vector consisting of acoustic and textual features, and eventually trained an SVM classifier with linear kernel. In order to extract acoustic features, they used i-vectors and x-vectors, while for extracting the textual features they employed a BERT model. Results showed that the early fusion increased the classification results obtained by training unimodal models, achieving an accuracy of 81.25%.

Sarawgi et al. (2020) proposed three individual acoustic and textual models, namely disfluency, interventions, and acoustic. For classifying subjects into AD and non-AD patients, the authors exploited the outputs of the individual models *via* different kinds of ensemble methods, namely hard, soft, and learnt ensemble. Regarding the hard ensemble, it corresponded to a majority vote of the predictions made by the individual models, while the soft ensemble corresponded to a weighted sum of the class probabilities. In terms of the learnt ensemble, the authors used a Logistic Regression to learn the weights in contrast to the soft ensemble, where the confidence of each model was treated equally. Results suggested that the hard ensemble was the best performing approach achieving an accuracy of 83.00%. For predicting the MMSE scores, the authors averaged the predictions of the individual models.

Also, Cummins et al. (2020) introduced a fusion approach for predicting AD patients. For the audio modality, the authors proposed a bag-of-audio-words approach, a siamese network trained with Log-Mel spectrograms, and an end-to-end convolutional neural network trained with raw audio waveforms. In terms of the textual modality, the authors used the GloVe embeddings and trained a bi-directional Hierarchical Attention Network (bi-HANN), a bi-directional LSTM (bi-LSTM), and a bi-directional LSTM with an attention mechanism (bi-LSTM-Att). After employing several acoustic and textual individual models, they stated that an accuracy of 85.20% was obtained *via* a majority vote of the three best performing models, two of which are acoustic models and the one corresponds to the textual model. Regarding the MMSE regression task, the best result of RMSE equal to 4.65 was achieved *via* a weighted average (fusion) of three approaches.

Martinc and Pollak (2020) also introduced an early fusion approach between different types of audio and textual features to detect AD patients and predict the MMSE scores. The authors trained four distinct classification algorithms, namely XGBoost, Random Forest, SVM with linear kernel, and Logistic Regression. Results suggested that Logistic Regression and SVMs with linear kernels were proved better than XGBoost and Random Forest models. Also, the readability of the transcript and the duration of the audio files were proved to be two of the best features.

Shah et al. (2021) used also an ensemble method to predict AD patients. Specifically, after training acoustic and language models, they chose the three best performing acoustic models and the best performing language model. Then, the authors computed a final prediction by taking a linear weighted combination of the individual model predictions. The authors claimed that the weighted majority vote approach enhanced the performance of the individual models achieving an accuracy of 81.00%. Regarding the MMSE regression task, the authors computed an unweighted averaging of the best language and acoustic model predictions for MMSE scores.

Syed et al. (2021) proposed several acoustic and language individual models. Specifically, they extracted both handcrafted features and embeddings *via* BERT, RoBERTa, VGGish, YAMNet, etc. After applying feature aggregation techniques, they trained and tested a Logistic Regression and Support Vector Machine classifier for differentiating AD from non-AD patients. For fusing the two modalities, the authors applied a majority voting based label fusion strategy, where each model made a decision on whether it considered the subject to be healthy or suffering from Alzheimer's dementia. Results showed that the multimodal fusion did not achieve better performance than the unimodal models. Regarding the MMSE regression task, the authors used SVR and PLSR and fused the two modalities by applying average-based fusion.

Mittal et al. (2021) applied a late fusion strategy, where the output probabilities of the individual textual and acoustic models were combined in a weighted manner, and a threshold was fixed for classifying the persons into AD and healthy control (HC). Results indicated that the proposed approach achieved comparable results to the state-of-the-art ones.

Pappagari et al. (2021) proposed several acoustic and language models and fused the two modalities by using the output probabilities of the individual models as the inputs to a Logistic Regression classifier for obtaining a final prediction. Regarding the acoustic models, they used an end-to-end classifier by fine-tuning an x-vector model and trained a Logistic Regression and XGBoost classifier using features extracted *via* several open-source libraries. Regarding the language models, they fine-tuned a BERT model. Findings suggested that the fusion of the two modalities yielded an accuracy of 84.51%. Regarding the MMSE regression task, the linguistic approach by fine-tuning a BERT model obtained the lowest RMSE score accounting for 3.85.

Similarly, Pappagari et al. (2020) extracted a set of acoustic and linguistic features. In order to fuse the two modalities and obtain the predictions on the test set, they employed the scores from the whole training subset to train a final fusion GBR model that was used to perform the fusion of scores coming from the acoustic and transcript-based models for the challenge evaluation. Results showed that the fusion of acoustic and language models achieved the highest accuracy accounting for 75.00%. With regards to the MMSE regression task the authors averaged the scores from the different models.

On the other hand, Rohanian et al. (2020) extracted a set of textual and acoustic features and proposed a multimodal model with a gating mechanism (Srivastava et al., 2015). Specifically, their introduced model consisted of two branches of Bi-LSTM, one branch for each modality. The outputs of the respective branches were fed into a gating mechanism, so as to control the influence of each modality toward the final classification. Results indicated that the incorporation of the gating mechanism enhanced the classification performance yielding an accuracy of 79.20% and an RMSE score of 4.54 on the ADReSS Challenge test set. Similarly, Rohanian et al. (2021) replaced the branch of BiLSTMs corresponding to the textual modality with BERT. Results showed that BiLSTM outperformed BERT and the authors speculated that this may be attributable to the fact that BERT is very large in comparison to the LSTM models.

Edwards et al. (2020) introduced a different approach by transcribing the segment text into phoneme written pronunciation using CMUDict (Weide, 2005) and training several text classifiers on these representations with FastText obtaining the highest classification performance. Regarding the acoustic modality, after extracting several features and applying feature selection techniques, the authors stated that the ComParE2016 feature set is the best among the proposed acoustic feature sets. Finally, they claimed that the combination of phonemes and audio features achieved an accuracy of 79.17%.

Koo et al. (2020) introduced a novel architecture consisting of attention, CNN, BiLSTM, and dense layers. The authors extracted several features from the textual and acoustic modalities and they incorporated them in the introduced architecture. Specifically, for the audio modality, they extracted features using the openSMILE v2.3 toolkit (Eyben et al., 2010) and employed also the VGGish. Regarding the textual modality, they used handcrafted features, transformer-based embeddings, and GloVe embeddings. They trained several models using different sets of features and stated that the auditory information led to some performance degradation compared to the textual one. A majority vote on the predictions made by five individual neural networks achieved an accuracy of 81.25%. In terms of the MMSE regression task, the final prediction was taken as the median value. The lowest RMSE score was equal to 3.75 and was achieved by using both acoustic and textual modalities.

Zhu et al. (2021) employed both acoustic and language models and introduced multimodal approaches. Regarding the acoustic models, they exploited YAMNet, MobileNet, and Speech BERT. In terms of the language models, they employed BERT and Longformer. With regards to the multimodal models, the authors added or concatenated the textual and acoustic representations, thus treating equally each modality. Results suggested that the concatenation of the representations obtained by BERT and Speech BERT achieved the highest accuracy accounting for 82.92%.

Mahajan and Baths (2021) introduced a novel architecture consisting of CNN, BiLSTM, Attention, and Dense layers. The authors concatenated the acoustic and language embeddings obtained *via* branches of the proposed architecture and used a dense layer with two units to differentiate AD from non-AD patients. Finally, the authors stated that the proposed architecture reached accuracy up to 72.92% on the test set.

Balagopalan et al. (2021) compared the performance of traditional machine learning classifiers with the performance obtained by pre-trained transformer models, namely BERT. More specifically, the authors extracted a large number of features, i.e., lexicosyntactic, semantic, and acoustic features and applied feature selection by choosing top-k number of features, based on ANOVA *F*-value between label and features. Four conventional machine learning models, namely Support Vector Machine, Neural Network, Random Forest, and Naive Bayes, were trained with the respective sets of features. Next, the authors trained a BERT model and stated that BERT outperformed the feature-based approaches in terms of all the evaluation metrics.

Farzana and Parde (2020) introduced some approaches to predict MMSE scores using textual and acoustic features. More specifically, the authors extracted lexicosyntactic features weighted *via* tf-idf, psycholinguistic features, discourse-based features, and acoustic features (MFCCs). The authors trained a Support Vector Regressor for predicting the MMSE scores. Results indicated that a selection of verbal and non-verbal cues achieved the lowest RMSE score.

### 2.3. Related Work Review Findings

From the aforementioned research works it is evident that existing research initiatives use mainly early or late fusion and ensemble strategies, in order to detect AD patients and predict the MMSE scores. Furthermore, they use the add or the concatenation operation for fusing the representations of the different modalities. Thus, research works fail to model the interactions between the different modalities and control the influence of each one of them toward the final prediction.

Therefore, our work differs significantly from the aforementioned research works, since we *(a)* exploit BERT and ViT for extracting the textual and visual representations, respectively, and employ a co-attention layer at the top of the proposed architecture, *(b)* introduce an architecture, which injects visual and acoustic information to a BERT model *via* an Attention Gate, which controls the importance of the different modalities, and *(c)* introduce an architecture, which includes a variant of the self-attention mechanism and aims to capture the inter- and intra-modal interactions.

## 3. Materials and Methods

### 3.1. Dataset

We use the ADReSS Challenge dataset (Luz et al., 2020) for conducting our experiments. We choose this dataset, since it has been selected in a way so as to minimize various kinds of biases in the prediction task. Specifically, it is balanced for gender and age. Concurrently, it aims to mitigate common biases often overlooked in evaluations of AD detection methods, including repeated occurrences of speech from the same participant, which is common in longitudinal datasets, and variations in audio quality. Moreover, in contrast to other datasets, it is balanced, since it includes 78 AD and 78 non-AD patients. The ADReSS Challenge dataset has been split into a train and a test set. The train set consists of 108 people, where 54 people are AD patients and 54 people are non-AD ones. The test set comprises 48 people, where 24 people are AD patients and 24 people are non-AD ones.

### 3.2. Tasks

#### 3.2.1. AD Classification Task

Let a labeled dataset consist of transcripts and their corresponding audio files belonging to AD patients and non-AD ones. Transcripts belonging to AD subjects are given the label 1, while transcripts belonging to the non-AD patients are given the label 0. The task is to identify, if a transcript along with its audio file belongs to a person suffering from dementia, or to a person belonging to the healthy control group (binary classification problem).

#### 3.2.2. MMSE Regression Task

Let a dataset consist of transcripts and their corresponding audio files belonging to AD patients and non-AD ones. Each transcript along with the audio file has been assigned with a MMSE score ranging from 0 to 30, where a MMSE score of 25–30 is considered as normal, a MMSE score of 21–24 as mild, a MMSE score of 10–20 as moderate, and a MMSE score less than 10 as severe impairment (Rohanian et al., 2020). Given the transcript and the audio file, the task is to predict the MMSE score (regression problem).

### 3.3. Predictive Models

In this section, we present the proposed predictive models for detecting dementia using speech and transcripts. We use the python library PyLangAcq (Lee et al., 2016) for having access to the manual transcripts, since the dataset has been created using the CHAT (MacWhinney, 2000) coding system. Moreover, we employ the Python library librosa (McFee et al., 2021) for converting the audio files to Log-Mel spectrograms, their delta, and delta-delta (acceleration values). For all the experiments conducted, we use 224 Mel bands, hop length equal to 1,024, and a Hanning window. Each image is resized to (224 × 224) pixels.

#### 3.3.1. BERT + ViT + Co-attention

We pass the transcripts through a BERT model (Vaswani et al., 2017; Devlin et al., 2019) and the corresponding images through a ViT model (Dosovitskiy et al., 2020). Then, we use a co-attention mechanism (Lu et al., 2016; Shu et al., 2019) over the outputs of the aforementioned models, since it can help learn the attention weights of transcripts and image patches concurrently.

Formally, let *C* ∈ ℝ^*d*×*N*^ and *S* ∈ ℝ^*d*×*T*^ be the outputs of the BERT and ViT pretrained models, respectively. Following the methodology proposed by Lu et al. (2016), given the output of the BERT (**C** ∈ ℝ^*d*×*N*^) and the output of the ViT (**S** ∈ ℝ^*d*×*T*^), where *d* denotes the hidden size of the model, *N* and *T* the sequence length of the transcripts and image patches, respectively, the affinity matrix *F* ∈ ℝ^*N*×*T*^ is calculated using the equation presented below:


(1) $$\begin{array}{l} F=\tanh\left(C^{T}W_{l}S\right) \end{array}$$

where $W_{l}\in {\mathbb{R}}^{d\times d}$ is a matrix of learnable parameters. Next, this affinity matrix is considered as a feature and we learn to predict the transcript and image attention maps *via* the following,


(2) $$\begin{array}{l} H^{s}=\tanh\left(W_{s}S+\left(W_{c}C\right)F\right) \end{array}$$

(3) $$\begin{array}{l} H^{c}=\tanh\left(W_{c}C+\left(W_{s}S\right)F^{T}\right) \end{array}$$

where $W_{s},W_{c}\in {\mathbb{R}}^{k\times d}$ are matrices of learnable parameters. The attention probabilities for each word in the transcripts and each image patch are calculated through the softmax function as follows,


(4) $$\begin{array}{l} a^{s}=softmax\left(w_{hs}^{T}H^{s}\right) \end{array}$$

(5) $$\begin{array}{l} a^{c}=softmax\left(w_{hc}^{T}H^{c}\right) \end{array}$$

where $a_{s}\in {\mathbb{R}}^{1\times T}$ and $a_{c}\in {\mathbb{R}}^{1\times N}$. $w_{hs},w_{hc}\in {\mathbb{R}}^{k\times 1}$ are the weight parameters. Based on the above attention weights, the attention vectors for text and image representations are obtained *via* the following equations:


(6) $$\begin{array}{l} ŝ=\overset{T}{\underset{i=1}{\sum }}a_{i}^{s}s^{i},\;ĉ=\overset{N}{\underset{j=1}{\sum }}a_{j}^{c}c^{j} \end{array}$$

where ŝ ∈ ℝ^1×*d*^ and ĉ ∈ ℝ^1×*d*^.

Finally, these two vectors are concatenated.

Regarding the **AD detection problem** described in section 3.2.1, the resulting vector (*p* ∈ ℝ^1×2*d*^) is passed to a dense layer with 128 units and a ReLU activation function followed by a dense layer consisting of two units.

Regarding the **MMSE prediction problem** described in section 3.2.2, the resulting vector (*p* ∈ ℝ^1×2*d*^) is passed to a dense layer with 128 units and a ReLU activation function followed by a dense layer consisting of one unit with a ReLU activation function.

**Figure 1:**
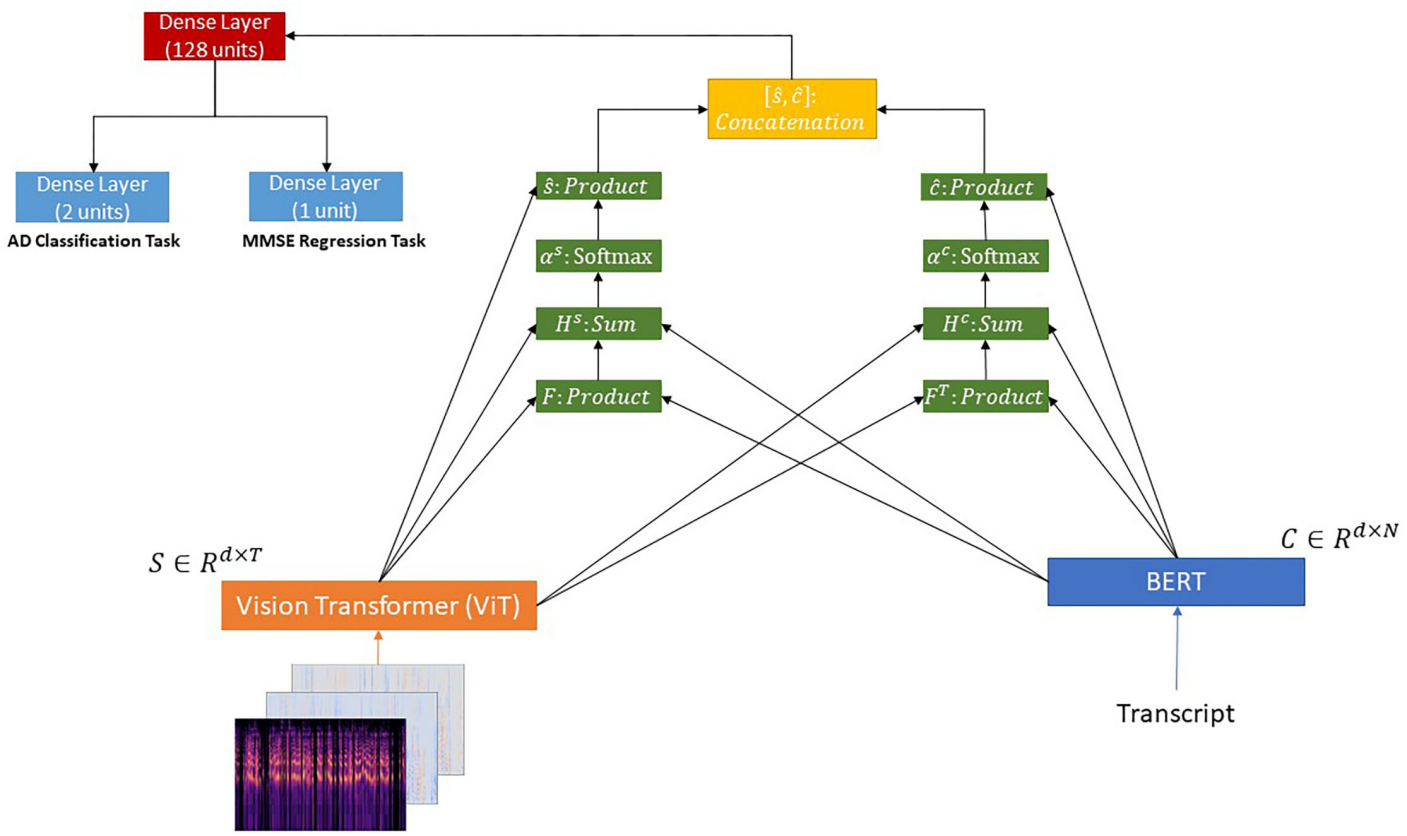
BERT + ViT + Co-Attention.

The proposed architecture is illustrated in Figure 1.

#### 3.3.2. Multimodal BERT

In this section, we introduce a similar methodology to the one adopted by Wang et al. (2019), Jin and Aletras (2020, 2021), and Rahman et al. (2020). First, we pass each transcript through a BERT model obtaining a text representation *X* ∈ ℝ^*N*×*d*^. Similarly, we pass each image through a ViT model and get the output of the ViT model ($z_{0}^{L}\in {\mathbb{R}}^{1\times d}$). Then, we repeat the vector $z_{0}^{L}$ *N* times, in order that the text and image representation matrices have the same size. Regarding the acoustic modality, we use the Python library openSMILE (Eyben et al., 2010) for extracting the eGeMAPSv02 feature set per audio file. We obtain a vector of 88d per audio file, where we project the respective vector to a 256d vector and repeat it *N* times. Let $e^{\left(i\right)},h_{\alpha }^{\left(i\right)},$ and $h_{v}^{\left(i\right)}$ denote word, acoustic, and image representation for the *i*-th word in a sequence. Next, we concatenate the representations (text-image and text-audio) using two attention gating mechanisms as described *via* the equations below:


(7) $$\begin{array}{l} w_{v}^{\left(i\right)}=\sigma \left(W_{hv}\left[h_{v}^{\left(i\right)};e^{\left(i\right)}\right]+b_{v}\right) \end{array}$$

(8) $$\begin{array}{l} w_{\alpha }^{\left(i\right)}=\sigma \left(W_{h\alpha }\left[h_{\alpha }^{\left(i\right)};e^{\left(i\right)}\right]+b_{\alpha }\right) \end{array}$$

where σ denotes the sigmoid activation function, *W*_*hv*_, *W*_*hα*_ are two weight matrices, and $w_{v}^{\left(i\right)},w_{\alpha }^{\left(i\right)}$ correspond to the visual and acoustic gates, respectively. *b*_*v*_ and *b*_α_ are the scalar biases.

Next, we calculate a non-verbal shift vector $h_{m}^{\left(i\right)}$ by multiplying the visual embeddings with the visual gate and the acoustic embeddings with the acoustic gate.


(9) $$\begin{array}{l} h_{m}^{\left(i\right)}=w_{v}^{\left(i\right)}\cdot \left(W_{v}h_{v}^{\left(i\right)}\right)+w_{\alpha }^{\left(i\right)}\cdot \left(W_{\alpha }h_{\alpha }^{\left(i\right)}\right)+b_{m}^{\left(i\right)} \end{array}$$

where *W*_*a*_ and *W*_*v*_ are weight matrices for acoustic and visual information, respectively. $b_{m}^{\left(i\right)}$ is the bias vector.

Next, we apply the Multimodal Shifting component aiming to dynamically shift the word representations by integrating the non-verbal shift vector $h_{m}^{\left(i\right)}$ into the original word embedding.


(10) $$\begin{array}{l} e_{m}^{\left(i\right)}=e^{\left(i\right)}+\alpha h_{m}^{\left(i\right)} \end{array}$$

(11) $$\begin{array}{l} \alpha =min\left(\frac{||e^{\left(i\right)}|{|}_{2}}{||h_{m}^{\left(i\right)}|{|}_{2}}\beta ,1\right), \end{array}$$

where β is a hyperparameter. Then, we apply a layer normalization (Ba et al., 2016) and dropout layer (Srivastava et al., 2014) to $e_{m}^{\left(i\right)}$. Finally, the combined embeddings are fed to a BERT model.

Regarding the **AD detection problem** described in section 3.2.1, the CLS token constituting the output of the BERT model is passed through a dense layer with 128 units and a ReLU activation function followed by a dense layer with two units, which gives the final output.

Regarding the **MMSE prediction problem** described in section 3.2.2, the CLS token constituting the output of the BERT model is passed through a dense layer with 128 units and a ReLU activation function followed by a dense layer with one unit and a ReLU activation function.

We experiment with injecting acoustic information **(Multimodal BERT - eGeMAPS)**, visual information **(Multimodal BERT - ViT)**, and both acoustic and visual information **(Multimodal BERT - eGeMAPS + ViT)**.

**Figure 2:**
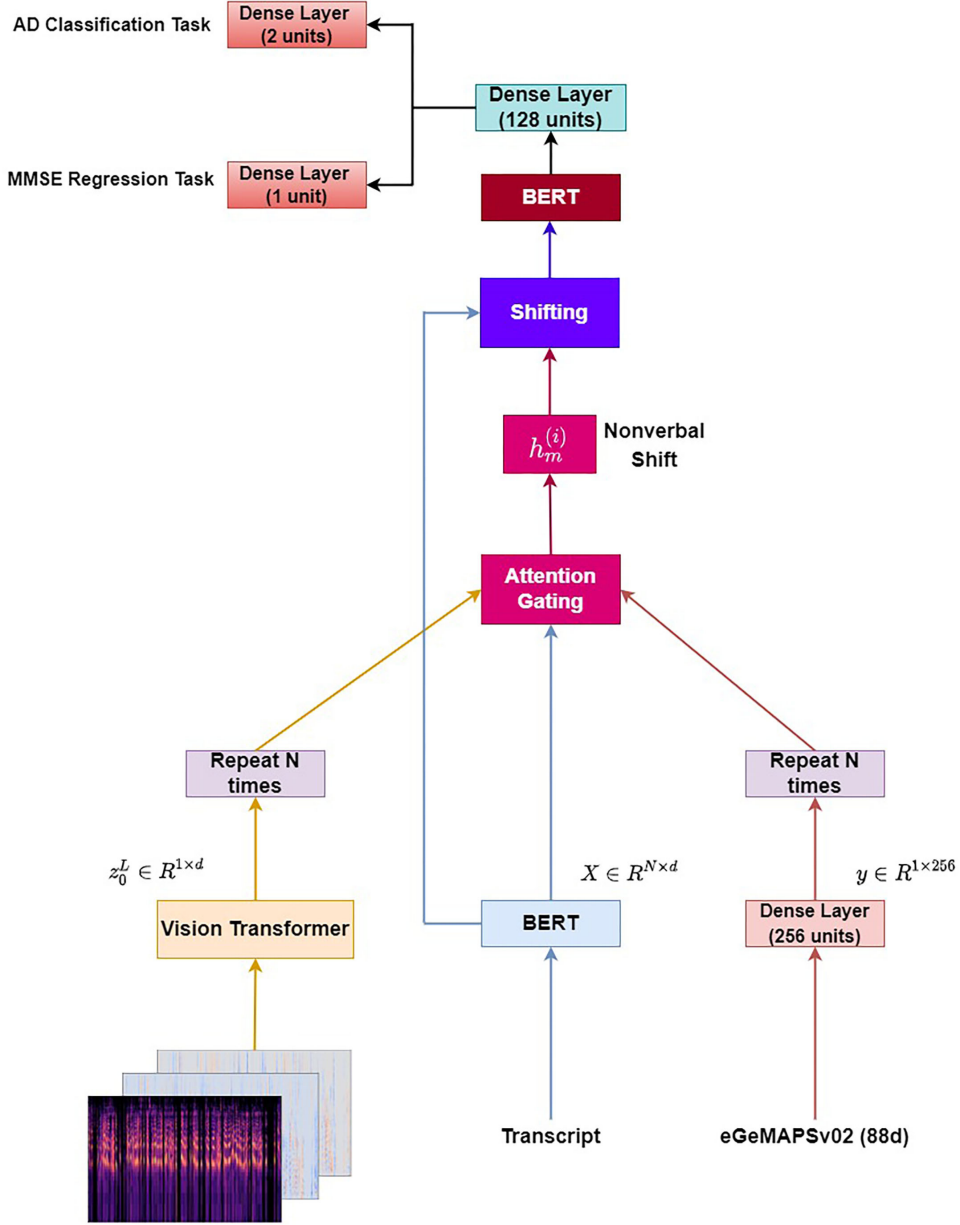
Multimodal BERT - eGeMAPS + ViT.

The architecture **(Multimodal BERT - eGeMAPS + ViT)** is illustrated in Figure 2.

#### 3.3.3. BERT + ViT + Gated Self-Attention

Similar to the aforementioned introduced models, we pass each transcript through a BERT model and each image through a ViT model. Let *X* ∈ ℝ^*N*×*d*^ and *Y* ∈ ℝ^*T*×*d*^ be the outputs of the BERT and ViT pretrained models, respectively. In this section, our main aim is to model the intra-modal and inter-modal interactions at the same time (i.e., *X* → *X*, *Y* → *Y*, and *X* ↔ *Y*). Thus, we adopt the methodology introduced by Yu et al. (2019).

After having obtained *X* ∈ ℝ^*N*×*d*^ and *Y* ∈ ℝ^*T*×*d*^, which correspond to the text and image representations, respectively, we concatenate these two representations as follows:


(12) $$\begin{array}{l} Z=\left[X;Y\right] \end{array}$$

Next, *Z* ∈ ℝ^*m*×*d*^, where *m* = *N* + *T*, is considered the query *Q*, key *K*, and value *V*, as follows:


(13) $$\begin{array}{l} Q=Z,K=Z,V=Z \end{array}$$

Next, we adopt the gating model introduced by Yu et al. (2019) as follows:


(14) $$\begin{array}{l} M=\sigma \left(FC^{g}\left(FC_{q}^{g}\left(Q\right)⊙FC_{k}^{g}\left(K\right)\right)\right) \end{array}$$

where $FC_{q}^{g},FC_{k}^{g}\in {\mathbb{R}}^{d\times d_{g}}$, $FC^{g}\in {\mathbb{R}}^{d_{g}\times 2}$ are three fully-connected layers, and *d*_*g*_ denotes the dimensionality of the projected space. ⊙ denotes the element-wise product function and σ the sigmoid function. In addition, *M* ∈ ℝ^*m*×2^ corresponds to the two masks $M_{q}\in {\mathbb{R}}^{m}$ and $M_{k}\in {\mathbb{R}}^{m}$ for the features *Q* and *V*, respectively.

Next, the two masks *M* and *K* are tiled to $\tilde{M_{q}},\tilde{M_{k}}\in {\mathbb{R}}^{m\times d}$ and then used for computing the attention map as following:


(15) $$\begin{array}{l} A^{g}=softmax\left(\frac{\left(Q⊙\tilde{M_{q}}\right){\left(K⊙\tilde{M_{k}}\right)}^{T}}{\sqrt{d}}\right) \end{array}$$

(16) $$\begin{array}{l} H=A^{g}V \end{array}$$

Then, the output *H* is passed through a global average pooling layer followed by a dense layer with 128 units and a ReLU activation function.

Regarding the **AD detection problem** described in section 3.2.1, we use a dense layer with two units, which gives the final output.

Regarding the **MMSE prediction problem** described in section 3.2.2, we use a dense layer with one unit and a ReLU activation function.

**Figure 3:**
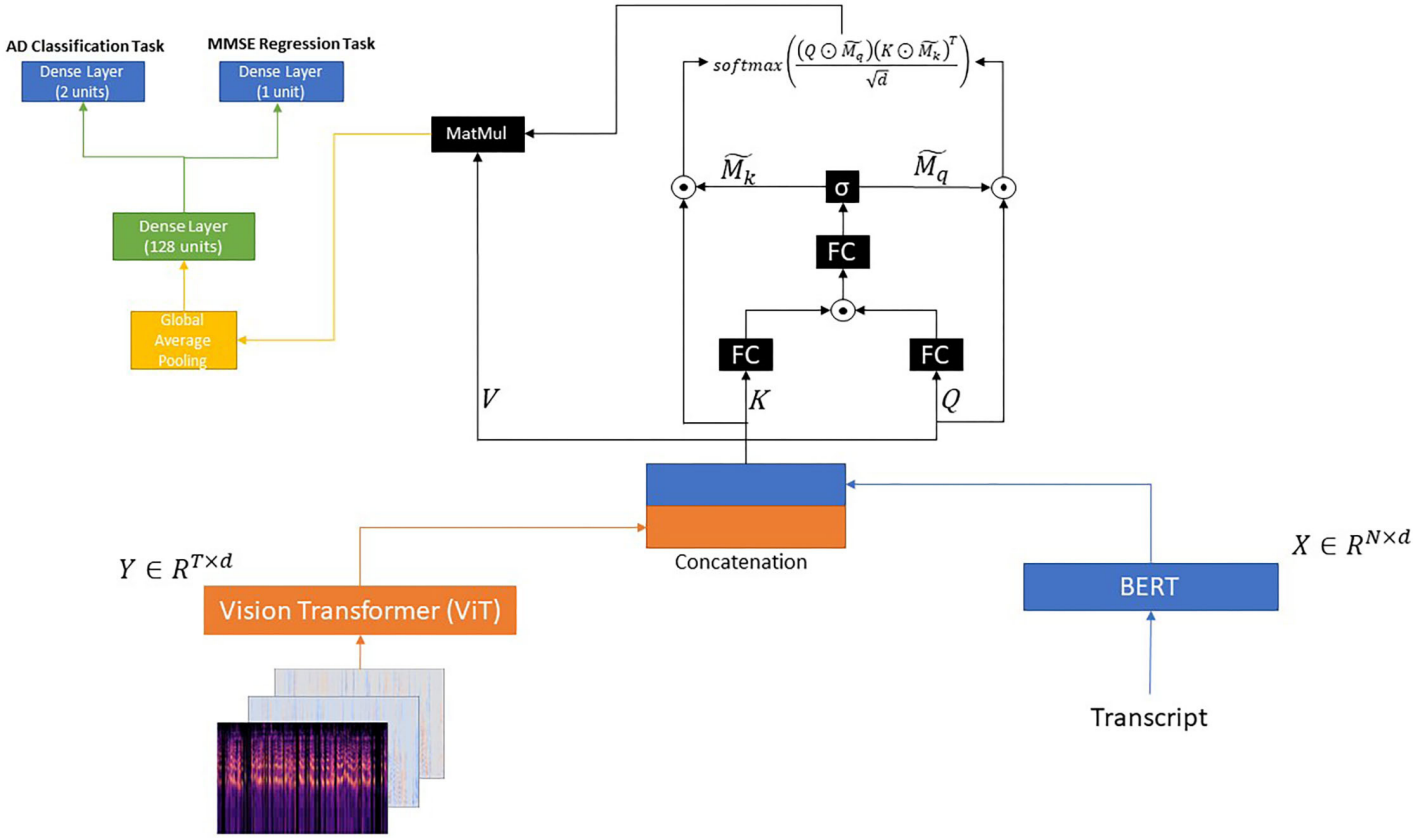
BERT + ViT + Gated Self-Attention.

The proposed architecture is illustrated in Figure 3.

## 4. Experiments

### 4.1. Comparison With State-of-the-Art Approaches

We compare our introduced models with research works proposing either unimodal or multimodal approaches. These research works have been selected due to the fact that they conduct their experiments on the ADReSS Challenge test set. These research works are reported in Tables 1–3. More specifically, Table 1 refers to research works using multimodal approaches, Table 2 refers to research works proposing unimodal approaches using only text, and Table 3 refers to research works proposing unimodal approaches using only speech.

**Table 1:** Overview of the multimodal state-of-the-art approaches, which are later compared with our work.

**References**	**Architecture**	**Features/methodology**	**Task**
Cummins et al. (2020)	Fusion Maj./W-avg (3-best)	Bag-of-Audio-Words, zero-frequency filtered (ZFF) signals, and BiLSTM-Attention network	AD/MMSE
Rohanian et al. (2020)	LSTM with Gating (Acoustic + Lexical + Dis)	Acoustic, Linguistic Features, Bi-LSTM, gating mechanism	AD/MMSE
Edwards et al. (2020)	System 3: Phonemes and Audio	Phoneme written pronunciation using CMUDict + acoustic features	AD
Pompili et al. (2020)	Fusion of System	Fusion of x-vectors with linguistic features, train SVM	AD
Koo et al. (2020)	Bimodal Network (Ensembled Output)	Ensemble (top-5 bimodal networks)	AD/MMSE
Martinc and Pollak (2020)	GFI, NUW, Duration, Character 4-grams, Suffixes, POS tag, UD	Feature extraction, Logistic Regression Classifier	AD
Pappagari et al. (2020)	Acoustic & Transcript	Fusion of the acoustic (x-vectors) and transcript (BERT) model scores	AD
Pappagari et al. (2020)	Acoustic+silence & Transcript	Average the scores from the different models, four silence features	MMSE
Zhu et al. (2021)	Dual BERT	Concatenation of the representations obtained by BERT and Speech BERT	AD
Mahajan and Baths (2021)	Model C	Neural network consisting of CNN, BiLSTM, Attention, GRU, and Dense layers	AD
Shah et al. (2021)	Majority vote (NLP + Acoustic)	Final prediction by taking a linear weighted combination of the individual model predictions	AD
Shah et al. (2021)	Random Forest (NLP) + gradient boosting (acoustic)	Language/fluency/n-gram features, MFCC and delta coefficients, Dimensionality Reduction Techniques	MMSE
Syed et al. (2021)	Audio + Text	Majority level approach of six models, averaging-based fusion	AD/MMSE
Sarawgi et al. (2020)	Ensemble	Majority voting approach, average the predictions	AD/MMSE
Syed et al. (2020)	Attempt 4	Label fusion from the top-5 performing models from audio and text modalities (top-5 from each modality), average value of predictions of individual models	AD/MMSE
Farzana and Parde (2020)	SELECTED-FEATURE	For selecting the features, a Random Forest regression model was trained. The authors retained only features having mean decrease impurity (MDI) values exceeding a predefined threshold	MMSE

**Table 2:** Overview of the unimodal state-of-the-art approaches using only text, which are later compared with our work.

**References**	**Architecture**	**Features/methodology**	**Task**
Cummins et al. (2020)	bi-LSTM-Att	GloVe 100d as pretrained weights, maximum word number for each transcript is 200, Bi-LSTM with attention	AD/MMSE
Rohanian et al. (2020)	LSTM (Lexical + Dis)	GloVe features of 100d, disfluency markers (self-repair), Bi-LSTM	AD/MMSE
Edwards et al. (2020)	System 2: Phonemes	The authors transcribed the segment text into phoneme written pronunciation using CMUDict. FastText was trained on the phoneme representation	AD
Pompili et al. (2020)	Sentence Embedding	Sentence embeddings are computed by averaging the second to twelfth hidden layers of each word., train SVM	AD
Koo et al. (2020)	Transformer-XL	The authors extracted textual features using Transformer-XL and trained a neural network consisting of CNN, Attention, Bi-LSTM, and Dense Layers.	AD/MMSE
Pappagari et al. (2020)	Transcript	The authors train a BERT model.	AD/MMSE
Zhu et al. (2021)	Longformer	Training of Longformer	AD
Mahajan and Baths (2021)	Model A0	Neural network consisting of CNN, LSTM, and Dense layers	AD
Shah et al. (2021)	Logistic Regression (NLP)	Language and fluency features, n-gram features, Dimensionality Reduction Techniques	AD
Shah et al. (2021)	Random Forest (NLP)	Language and fluency features, n-gram features, Dimensionality Reduction Techniques	MMSE
Syed et al. (2021)	Text (fusion)	Fusion of top-3 performing models from the textual modality	AD/MMSE
Syed et al. (2020)	Attempt 5	Label fusion from the top-10 performing models from text modalities, average of MMSE score predictions from the top-10 performing models	AD/MMSE
Balagopalan et al. (2021)	BERT	Training of BERT model	AD
Farzana and Parde (2020)	n-gram	All lexicosyntactic features, SVR training	MMSE
Meghanani et al. (2021b)	fastText, bi+trigram	The authors fit 21 models and the outputs are combined by a majority voting scheme for final classification. In the regression task, the outputs of these bootstrap models are averaged to arrive at the final MMSE score	AD/MMSE

**Table 3:** Overview of the unimodal state-of-the-art approaches using only speech, which are later compared with our work.

**References**	**Architecture**	**Features/methodology**	**Task**
Cummins et al. (2020)	SiameseNet	A deep Siamese neural network consisting of convolutional layers. As an input, the model used either 8-s or 16-s segments.	AD
Cummins et al. (2020)	BoAW fusion (3-best)	MelFrequency Cepstral Coefficient (MFCC), log-Mel, and the COMPARE acoustic feature set	MMSE
Rohanian et al. (2020)	LSTM (Acoustic)	Higher-order statistics of COVAREP features. Bi-LSTM training	AD/MMSE
Edwards et al. (2020)	System 1: Audio	LDA posterior probabilities of ComParE2016 features	AD
Pompili et al. (2020)	x-vectors_SRE	The authors use both the SRE and the Voxceleb models for the x-vectors framework. train SVM	AD
Koo et al. (2020)	VGGish	The authors used VGGish features and trained a neural network consisting of Attention Layer, CNN, Bi-LSTM, and Dense Layers.	AD/MMSE
Pappagari et al. (2020)	Acoustic + Silence	Silency features, x-vector PCA-transformed coefficients, Probabilistic Linear Discriminant Analysis (PLDA) for detection and Support Vector Regression (SVR) for MMSE prediction	AD/MMSE
Zhu et al. (2021)	YAMNet	The input of YAMNet is the Mel spectrogram from audio data with dimensions of (p, t, 1)	AD
Mahajan and Baths (2021)	Model B0 (emobase)	GRU taking in audio segment features and finally combining the features from the speech segments into a common vector	AD
Shah et al. (2021)	Majority vote (Acoustic)	Acoustic feature extraction across all speech segments, weighted majority vote classification on segments	AD
Shah et al. (2021)	Gradient Boosting (Acoustic)	MFCC 1–16 features and their delta coefficients from 26 Mel-bands	MMSE
Syed et al. (2021)	Audio (fusion)	Majority level approach of three acoustic models, averaging-based fusion	AD/MMSE
Chlasta and Wolk (2021)	DemCNN	Convolutional neural network for speech classification using the raw waveform	AD
Meghanani et al. (2021a)	CNN - LSTM (MFCC)	21 models are fitted using the above 21 bootstrap samples and the outputs are combined by a majority voting scheme for final classification.	AD
Meghanani et al. (2021a)	pBLSTM-CNN (log-Mel)	Bagging of 21 models by averaging the outputs.	MMSE
Farzana and Parde (2020)	acoustic-all	Mel Frequency Cepstral Coefficients (MFCCs), mean value, variance, etc.	MMSE
Syed et al. (2020)	Attempt 3	Label fusion from the top-5 performing models from the audio modality, prediction from the BERT base uncased RangePool	AD/MMSE

### 4.2. Experimental Setup

#### 4.2.1. Training and Evaluation—Implementation Details

In terms of the **MMSE regression task**, the ADReSS Challenge train set includes the MMSE scores for all the people except one. Thus, we remove this person from the train set in the **MMSE regression task**.

We follow a similar training strategy to the one adopted by Zhu et al. (2021). Firstly, we divide the train set provided by the Challenge into a train and a validation set (65–35%). Next, we train the proposed architectures five times with an Adam optimizer and a learning rate of 1e-5. Regarding the **AD detection problem** described in section 3.2.1, we minimize the cross-entropy loss function, whereas with regards to the **MMSE prediction problem** described in section 3.2.2, we minimize the RMSE. We apply *ReduceLROnPlateau*, where we reduce the learning rate by a factor of 0.1, if the validation loss has stopped decreasing for three consecutive epochs. Also, we apply *EarlyStopping* and stop training, if the validation loss has stopped decreasing for six consecutive epochs. We test the proposed models using the test set provided by the Challenge. We average the results obtained by the five repetitions. All models have been created using the PyTorch library (Paszke et al., 2019). We have used the Vision Transformer (with fixed-size patches of resolution 16 × 16) and the BERT base uncased version from the Transformers library (Wolf et al., 2020). The input to the BERT and ViT model is the output of the BERT tokenizer and ViT feature extractor, respectively, as defined by the Transformers library. All experiments are conducted on a single Tesla P100-PCIE-16GB GPU.

#### 4.2.2. Hyperparameters

Regarding **BERT+ViT+Co-Attention**, we set *k* equal to 40. We use dropout after the output of the co-attention layer with a rate of 0.4, and a dropout layer after the dense layer consisting of 128 units with a rate of 0.2. Regarding **(Multimodal BERT - eGeMAPS)**, we set β = 0.01. In terms of **(Multimodal BERT - ViT)**, we set β = 0.001. Regarding **(Multimodal BERT - eGeMAPS + ViT)**, we set β = 0.01. With regards to the following models: **(Multimodal BERT - eGeMAPS)**, **(Multimodal BERT - ViT)**, and **(Multimodal BERT - eGeMAPS + ViT)**, we apply dropout with a rate of 0.4 at the output of (10) and freeze the weights of the first BERT model. Also, we use a dropout layer after the output of the second BERT model with a rate of 0.2. With regards to **BERT+ViT+Gated Self-Attention**, we set *d*_*g*_ = 64. We use dropout after the global average pooling layer with a rate of 0.3. For all the experiments conducted, the hidden size of BERT and ViT denoted by *d* is equal to 768. Moreover, *N* = 512, since we pad each transcript to a maximum number of 512 tokens. *T* is equal to 197. Thus, *m* is equal to 709.

#### 4.2.3. Evaluation Metrics

Regarding the **AD detection problem** described in section 3.2.1, Accuracy, Precision, Recall, F1-Score, and Specificity have been used for evaluating the results of the introduced architectures. These metrics have been computed by regarding the dementia class as the positive one. We report the average and standard deviation of these metrics over five runs.

With regards to the **MMSE prediction problem** described in section 3.2.2, the RMSE has been used for evaluating the results of the introduced architectures. We report the average and standard deviation of the RMSE scores across five runs. The RMSE is the metric used in the baseline paper provided by the ADReSS challenge.

## 5. Results

### 5.1. AD Classification Task

The results of the proposed models mentioned in section 3.3 for the AD classification task are reported in Table 4. In addition, in this table we compare the results of our introduced models with research works proposing multimodal approaches, unimodal models using only text data, and unimodal approaches using only speech data.

**Table 4:** AD Classification Task: Performance comparison among proposed models and state-of-the-art approaches on the ADReSS Challenge test set.

**Architecture**	**Precision**	**Recall**	**F1-score**	**Accuracy**	**Specificity**
**State-of-the-art approaches (Multimodal)**					
Cummins et al. (2020)	-	-	85.40	85.20	-
Rohanian et al. (2020)	-	-	-	79.20	-
Edwards et al. (2020)	81.82	75.00	78.26	79.17	-
Pompili et al. (2020)	**94.12**	66.67	78.05	81.25	-
Koo et al. (2020)	89.47	70.83	79.07	81.25	-
Martinc and Pollak (2020)	-	-	-	77.08	-
Pappagari et al. (2020)	70.00	88.00	78.00	75.00	-
Zhu et al. (2021)	83.04 ± 3.97	83.33 ± 5.89	82.92 ± 1.86	82.92 ± 1.56	-
Mahajan and Baths (2021)	78.94	62.50	69.76	72.92	-
Shah et al. (2021)	-	-	-	83.00	-
Syed et al. (2021)	-	87.50	-	89.58	91.67
Sarawgi et al. (2020)	83.00	83.00	83.00	83.00	-
Syed et al. (2020)	-	-	-	79.17	-
**State-of-the-art approaches (only Text)**					
Cummins et al. (2020)	-	-	81.20	81.30	-
Rohanian et al. (2020)	-	-	-	72.90	-
Edwards et al. (2020)	80.95	70.83	75.56	77.08	-
Pompili et al. (2020)	82.35	58.33	68.29	72.92	-
Koo et al. (2020)	80.00	83.33	81.63	81.25	-
Pappagari et al. (2020)	69.00	83.00	75.00	72.92	-
Zhu et al. (2021)	88.14 ± 2.09	74.17 ± 5.53	80.44 ± 3.55	82.08 ± 2.83	-
Mahajan and Baths (2021)	76.47	54.16	63.41	68.75	-
Shah et al. (2021)	-	-	-	85.00	-
Syed et al. (2021)	-	**91.67**	-	**91.67**	91.67
Syed et al. (2020)	-	-	-	85.42	-
Balagopalan et al. (2021)	83.89	83.33	83.27	83.32	83.33
Meghanani et al. (2021b)	86.00	79.00	83.00	83.33	88.00
**State-of-the-art approaches (only Speech)**					
Cummins et al. (2020)	-	-	70.80	70.80	-
Rohanian et al. (2020)	-	-	-	66.60	-
Edwards et al. (2020)	58.62	70.83	64.15	60.42	-
Pompili et al. (2020)	54.17	54.17	54.17	54.17	-
Koo et al. (2020)	78.95	62.50	69.77	72.92	-
Pappagari et al. (2020)	70.00	58.00	63.00	66.70	-
Zhu et al. (2021)	64.40 ± 3.93	73.40 ± 8.82	68.60 ± 4.84	66.20 ± 4.79	-
Mahajan and Baths (2021)	65.21	62.50	63.82	64.58	-
Shah et al. (2021)	-	-	-	65.00	-
Syed et al. (2021)	-	83.33	-	81.25	79.17
Chlasta and Wolk (2021)	62.50	62.50	62.50	62.50	62.50
Meghanani et al. (2021a)	82.00	38.00	51.00	64.58	92.00
Syed et al. (2020)	-	-	-	64.58	-
**Proposed transformer-based models**					
*BERT+ViT+Co-Attention*	92.83 ± 6.39	81.67 ± 2.04	86.81 ± 3.37	87.50 ± 3.49	**93.33** ± 6.24
*Multimodal BERT - eGeMAPS*	74.51 ± 1.01	87.50 ± 6.45	80.35 ± 2.77	78.75 ± 2.04	70.00 ± 3.12
*Multimodal BERT - ViT*	73.91 ± 2.40	**91.67** ± 2.64	81.79 ± 1.72	79.58 ± 2.04	67.50 ± 4.08
*Multimodal BERT - eGeMAPS+ViT*	76.57 ± 3.74	89.17 ± 5.65	82.28 ± 3.49	80.83 ± 3.58	72.50 ± 5.65
*BERT+ViT+Gated Self-Attention*	90.87 ± 3.50	89.17 ± 2.04	**89.94** ± 1.36	90.00 ± 1.56	90.83 ± 4.08

*Reported values are mean ± standard deviation. Results are averaged across five runs. Best results per evaluation metric are in bold*.

Regarding our proposed models, one can observe from Table 4 that BERT+ViT+Gated Self-Attention outperforms all the introduced models in Accuracy and F1-score by a large margin of 2.50–11.25% and 3.13–9.59%, respectively. This can be justified by the fact that the Gated Self-Attention aims to capture both the intra- and inter-modal interactions. Specifically, BERT+ViT+Gated Self-Attention outperforms BERT+ViT+Co-Attention in accuracy by 2.50%, in Recall by 7.5%, and in F1-score by 3.13%. Despite the fact that BERT+ViT+Co-Attention obtains a high specificity score accounting for 93.33% outperforming BERT+ViT+Gated Self-Attention by 2.5%, BERT+ViT+Co-Attention attains a low F1-score accounting for 86.81%. On the contrary, BERT+ViT+Gated Self-Attention yields an F1-score of 89.94% outperforming BERT+ViT+Co-Attention by 3.13%. This means that BERT+ViT+Gated Self-Attention can detect better the AD patients than BERT+ViT+Co-Attention, where AD patients are misdiagnosed as non-AD ones. In addition, although BERT+ViT+Gated Self-Attention obtains lower results in Precision and Recall by other introduced models, it surpasses them in F1-score, which constitutes the weighted average of recall and precision. Regarding the Multimodal BERT models, one can observe that Multimodal BERT-ViT outperforms Multimodal BERT-eGeMAPS in accuracy by 0.83%, in recall by 4.17%, and in F1-score by 1.44%. We speculate that Multimodal BERT-ViT performs better than Multimodal BERT-eGeMAPS due to the usage of the Vision Transformer. Thus, the visual modality obtained *via* ViT seems to perform slightly better than the acoustic modality. In addition, we observe that the injection of both the acoustic and visual information enhances the performance of the models having just one modality, be it either the acoustic modality or the visual one. More specifically, Multimodal BERT-eGeMAPS+ViT surpasses Multimodal BERT-eGeMAPS and Multimodal BERT-ViT in accuracy by 2.08 and 1.25%, respectively. In comparison to the Multimodal BERT-eGeMAPS+ViT, BERT+ViT+Gated Self-Attention surpasses its performance in accuracy by 9.17%, in Precision by 14.30%, in F1-score by 7.66%, and in Specificity by 18.33%. Overall, BERT+ViT+Gated Self-Attention constitutes our best performing model, since it surpasses all the other introduced models in F1-score and Accuracy.

In comparison to the multimodal approaches, as one can easily observe from Table 4, BERT+ViT+Gated Self-Attention surpasses the state-of-the-art multimodal approaches in Recall by 1.17–26.67%, in F1-Score by 4.54–20.18%, and in Accuracy by 0.42–17.08%. These findings confirm our initial hypothesis that inter- and intra-modal interactions enhance the classification results obtained by approaches, which predict AD patients either by using majority voting on predictions of several individual models or adding/concatenating the text and image representations. In addition, although our best performing model outperforms *Audio+Text* (Syed et al., 2021) by a small margin of 0.42% in Accuracy and by a larger margin of 1.67% in Recall, it is worth mentioning that our proposed approach is more computational and time-efficient, since the method proposed by Syed et al. (2021) employs six models and eventually uses a majority vote approach. In terms of BERT+ViT+Co-Attention, it outperforms all the research works, except *Audio+Text* (Syed et al., 2021), in Accuracy by 2.30–14.58%. Also, it surpasses all the research works, except *Fusion of System* (Pompili et al., 2020) in Precision by 3.36–22.83%. Also, it surpasses all the research works in F1-score results by 1.41–17.05%, and in Specificity by 1.66%. It outperforms four research works out of the eight ones, which report Recall results by 6.67–19.17%. Thus, the co-attention mechanism can yield better performance than the results obtained by the research initiatives, since it can attend to transcripts and images simultaneously. Finally, with regards to the proposed Multimodal BERT models, it seems that they are rather complex for our limited dataset. However, results suggest that Multimodal BERT - eGeMAPS+ViT surpasses six research works in Accuracy by 1.63–7.91%, five research works in F1-score by 3.21–12.52%, all the research works in the Recall score by 1.17–26.67%, and one research work in the Precision score by 6.57%.

In comparison to the unimodal approaches using only text data, as one can easily observe from Table 4, the approach proposed by Syed et al. (2021) outperforms our best performing model in terms of accuracy, recall, and specificity by 1.67, 2.50, and 0.84%, respectively. However, our best performing model outperforms all the other approaches in accuracy by 4.58–17.10%, in Recall by 5.84–35.01%, in Precision by 2.73–21.87%, in F1-score by 6.67–26.53%, and in Specificity by 2.83–7.50%.

In comparison to the unimodal approaches using only speech data, as one can easily observe from Table 4, BERT+ViT+Gated Self-Attention outperforms the research initiatives in terms of Precision, Recall, F1-score, and Accuracy. More specifically, BERT+ViT+Gated Self-Attention surpasses the research works in Precision by 8.87–36.70%, in Recall by 5.84–51.17%, in F1-score by 19.14–38.94%, and in Accuracy by 8.75–35.83%. In addition, BERT+ViT+Co-Attention surpasses the research works in Precision by 10.83–38.66%, in F1-score by 16.01–35.81%, and in Accuracy by 6.25–33.33%. Additionally, Multimodal BERT - eGeMAPS, Multimodal BERT - ViT, and Multimodal BERT - eGeMAPS + ViT outperform all the research initiatives except (Syed et al., 2021) in terms of the accuracy score by a margin of 5.83–24.58%, 6.66–25.41%, and 7.91–26.66%, respectively.

It is obvious that the unimodal approaches exploiting only speech data achieve low evaluation results in comparison with unimodal approaches employing text data or multimodal models.

### 5.2. MMSE Regression Task

The results of the proposed models mentioned in section 3.3 for the MMSE regression task are reported in Table 5. In addition, in this table we compare the results of our introduced models with research works proposing multimodal approaches, unimodal models using only text data, and unimodal approaches using only speech data.

**Table 5:** MMSE Regression Task: performance comparison among proposed models and state-of-the-art approaches on the ADReSS Challenge test set.

**Architecture**	**RMSE**
**State-of-the-art approaches (Multimodal)**	
Cummins et al. (2020)	4.65
Rohanian et al. (2020)	4.54
Koo et al. (2020)	3.77
Pappagari et al. (2020)	5.32
Shah et al. (2021)	6.01
Syed et al. (2021)	4.47
Martinc and Pollak (2020)	5.06
Syed et al. (2020)	4.91
Farzana and Parde (2020)	4.34
**State-of-the-art approaches (only Text)**	
Cummins et al. (2020)	4.66
Rohanian et al. (2020)	4.88
Koo et al. (2020)	4.02
Pappagari et al. (2020)	5.86
Shah et al. (2021)	5.62
Syed et al. (2021)	3.74
Syed et al. (2020)	4.30
Farzana and Parde (2020)	4.61
Meghanani et al. (2021b)	4.87
**State-of-the-art approaches (only Speech)**	
Cummins et al. (2020)	6.45
Rohanian et al. (2020)	5.93
Koo et al. (2020)	5.08
Pappagari et al. (2020)	5.97
Shah et al. (2021)	6.67
Syed et al. (2021)	5.86
Meghanani et al. (2021a)	5.90
Farzana and Parde (2020)	6.42
Syed et al. (2020)	5.18
**Proposed Transformer-based models**	
*BERT+ViT+Co-Attention*	4.20 ± 0.47
*Multimodal BERT - eGeMAPS*	5.64 ± 0.11
*Multimodal BERT - ViT*	5.50 ± 0.30
*Multimodal BERT - eGeMAPS+ViT*	5.62 ± 0.12
*BERT+ViT+Gated Self-Attention*	**3.61** ± 0.48

*Reported values are mean ± standard deviation. Results are averaged across five runs. Best results are in bold*.

Regarding our proposed models, one can observe from Table 5 that BERT + ViT + Gated Self-Attention obtains the lowest RMSE score accounting for 3.61 followed by BERT + ViT + Co-Attention, whose RMSE score is equal to 4.20. Regarding Multimodal BERT - eGeMAPS, Multimodal BERT - ViT, and Multimodal BERT - eGeMAPS + ViT, it is obvious that these neural networks are complex for the MMSE regression task achieving RMSE scores equal to 5.64, 5.50, and 5.62, respectively.

In comparison to the multimodal approaches, as one can easily observe from Table 5, BERT + ViT + Gated Self-Attention, which constitutes our best performing model, improves the RMSE score obtained by the multimodal state-of-the-art approaches by 0.15–2.40. Regarding BERT + ViT + Co-Attention, it improves the RMSE scores of all the existing research initiatives, except *Bimodal Network (Ensembled Output)* (Koo et al., 2020), by 0.14–1.41. In terms of the Multimodal BERT - eGeMAPS, Multimodal BERT - ViT, and Multimodal BERT - eGeMAPS + ViT, it seems that these architectures are rather complex for the MMSE regression task improving the RMSE score of only one research work (Shah et al., 2021).

In comparison with the unimodal approaches exploiting only text data, one can easily observe from Table 5 that BERT + ViT + Gated Self-Attention performs better than the existing research initiatives improving the current RMSE score by 0.13-2.25. In addition, BERT + ViT + Co-Attention achieves comparable performance to existing research works outperforming all the existing research works, except *Transformer-XL* (Koo et al., 2020) and *Text (fusion)* (Syed et al., 2021), by 0.10-1.66. Finally, Multimodal BERT - ViT obtains lower RMSE score than the one obtained by Pappagari et al. (2020) and Shah et al. (2021).

In comparison with the unimodal approaches using only speech data, one can observe from Table 5 that BERT + ViT + Gated Self-Attention outperforms all the research initiatives by a large margin of 1.47–3.06. Similarly, BERT + ViT + Co-Attention obtains lower RMSE score than the scores achieved by all the research works. Specifically, the performance gain ranges from 0.48 to 2.47. Finally, Multimodal BERT - eGeMAPS, Multimodal BERT - ViT, and Multimodal BERT - eGeMAPS + ViT outperform all the state-of-the-art approaches, except *Attempt 3* (Syed et al., 2020) and *VGGish* (Koo et al., 2020), improving the RMSE score by 0.22–1.03, 0.36–1.17, and 0.24–1.05, respectively.

It is obvious that the research works exploiting only speech data obtain higher RMSE scores than the ones exploiting text data or the combination of text and speech data.

## 6. Discussion

The detection of dementia from spontaneous speech has emerged into a hot topic throughout the years due to the fact that it constitutes a time-effective procedure. Although dementia detection from speech is a hot topic and item of interest from several researchers around the world, there are still significant limitations that need to be addressed. The main limitation is pertinent to the way the different modalities, i.e., acoustic, visual, and textual, are combined in a single neural network. Research works having proposed multimodal methods tend to train separately acoustic, language, and visual models and then apply majority vote or average-based approaches for the AD classification and MMSE regression task, respectively. In addition, they tend to add or concatenate the representations obtained by the different modalities, thus treating equally each modality. Therefore, in this study, we aim to tackle the aforementioned limitations and propose three novel architectures, which combine the different modalities effectively achieving competitive performance to existing research initiatives.

From the results obtained in this study for the AD classification task, we found that:

- **Finding 1:** The incorporation of a co-attention mechanism, which can learn the attention weights for words and image patches simultaneously, outperforms the multimodal research initiatives except one in terms of the Accuracy score.
- **Finding 2:** We propose a method to inject visual and acoustic modalities along with the textual one into a BERT model *via* a Multimodal Shifting Gate. We experiment with injecting only visual information, only acoustic information, and their combination. Findings state that the injection of both modalities performs better than the injection of single modalities.
- **Finding 3:** We introduce an approach aiming to model both the inter- and intra-modal interactions at the same time and show that this approach is the best performing one among the introduced approaches.

From the results obtained in this study for the MMSE regression task, we found that:

- **Finding 4:** The incorporation of the co-attention mechanism at the top of the pretrained models, i.e., BERT and ViT, obtains low RMSE improving all the state-of-the-art approaches except (Koo et al., 2020; Syed et al., 2021).
- **Finding 5:** Multimodal BERT models do not perform well to the MMSE regression task. These architectures are rather complex for the limited dataset used in this study.
- **Finding 6:** BERT+ViT+Gated Self-Attention improves the RMSE score in the MMSE regression task by 0.13–3.06 obtaining a new state-of-the-art result. The ability of this architecture to perform well both in the AD classification task and in the MMSE regression task establishes the usefulness of this architecture for the dementia detection problem and indicates that both the inter- and intra-modal interactions are important.

Although the unimodal approach proposed by Syed et al. (2021) outperforms our best performing model in the AD classification task, our best performing model obtains better results in the MMSE regression task. In addition, our introduced model is more computationally and time-effective, since the approach by Syed et al. (2021) extracts embeddings by employing transformer networks, applies feature aggregation techniques, trains traditional machine learning algorithms, and finally applies a majority voting approach of the top-3 performing models. Regarding the multimodal approach proposed by Syed et al. (2021), it achieves lower evaluation results than the unimodal approach. We speculate that this degradation in performance is attributable to the fact that the majority-vote approach does not take the interactions between the different modalities into consideration.

One limitation of the present methods is the usage of a dataset with a limited number of samples available. To be more precise, the ADReSS Challenge dataset consists of 108 people in the train set and 48 people in the test set. However, as mentioned in section 3.1, in contrast to other datasets, this dataset has been created in such a way so as to mitigate different kinds of biases, which could otherwise influence the validity of the proposed approaches during the training and evaluation procedure. More specifically, this dataset is matched for gender and age. Concurrently, the recordings have been acoustically enhanced for controlling for variation caused by recording conditions, such as microphone placement.

## 7. Conclusion and Future Work

In this article, we introduced three novel multimodal neural networks for detecting dementia (AD classification task) and predicting the MMSE scores (MMSE regression task) from spontaneous speech. First, we proposed a model consisting of BERT, ViT, and a co-attention mechanism at the top of the proposed architecture, which is capable of attending to both the words and the image patches simultaneously. Results indicated that the proposed model achieved an accuracy of 87.50% in the AD classification task outperforming all the research works proposing multimodal approaches except one. Regarding the MMSE regression task, our proposed architecture achieved an RMSE score equal to 4.20. Secondly, we introduced a deep learning architecture, where we injected information from the visual and acoustic modalities along with the textual one into a BERT model and used an attention gate mechanism to control the importance of each modality. Results for the AD classification task suggested that the injection of both the acoustic and visual modalities enhanced the performance of the models achieved when using only either the acoustic or the visual modality along with the textual one. Finally, we introduced a transformer-based network, where we concatenated the representations obtained *via* BERT and ViT and passed the representation through a self-attention mechanism incorporating a novel gating mechanism. Findings showed that this introduced model was the best performing one on the ADReSS Challenge test set reaching Accuracy and F1-score up to 90.00 and 89.94%, respectively. In terms of the MMSE regression task, our best performing model obtained an RMSE score of 3.61 improving the state-of-the-art RMSE scores for the regression task of the ADReSS Challenge by 0.13–3.06.

Our introduced approaches have been conducted on the ADReSS Challenge dataset. This dataset includes a statistically balanced and acoustically enhanced set of recordings from spontaneous speech aiming to address the lack of standardization and consequently embed novel approaches into clinical practice. Our proposed approaches developed on both the AD classification and MMSE regression task can be used for the development of a remote tool or app, which will be capable of detecting AD patients and predicting the MMSE scores using spontaneous speech.

In the future, we plan to investigate more methods on how to combine the different modalities effectively. In addition, due to the fact that there are datasets, i.e., ADReSSo Challenge (Luz et al., 2021), where the manual transcripts are not available, one should apply automatic speech recognition (speech to text) methods. Therefore, one of our future plans is to apply our methods on automated transcripts for exploring differences in performance in comparison to the manual transcripts.

## Data Availability Statement

Publicly available datasets were analyzed in this study. This data can be found here: http://www.homepages.ed.ac.uk/sluzfil/ADReSS/.

## Ethics Statement

The studies involving human participants were reviewed and approved by DementiaBank Consortium. The patients/participants provided their written informed consent to participate in this study.

## Author Contributions

LI contributed to the conceptualization, design and implementation of the methodology, software development, analysis of the results, and writing of the manuscript. DA contributed to the design of the research and supervised the findings of the work. All authors contributed to the article and approved the submitted version.

## Conflict of Interest

The authors declare that the research was conducted in the absence of any commercial or financial relationships that could be construed as a potential conflict of interest.

## Publisher's Note

All claims expressed in this article are solely those of the authors and do not necessarily represent those of their affiliated organizations, or those of the publisher, the editors and the reviewers. Any product that may be evaluated in this article, or claim that may be made by its manufacturer, is not guaranteed or endorsed by the publisher.
